# Upper Aerodigestive Tract Squamous Cell Carcinomas Show Distinct Overall DNA Methylation Profiles and Different Molecular Mechanisms behind WNT Signaling Disruption

**DOI:** 10.3390/cancers13123014

**Published:** 2021-06-16

**Authors:** Sheila Coelho Soares-Lima, Hisham Mehanna, Diego Camuzi, Paulo Thiago de Souza-Santos, Tatiana de Almeida Simão, Pedro Nicolau-Neto, Monique de Souza Almeida Lopes, Cyrille Cuenin, Fazlur Rahman Talukdar, Nikolaos Batis, Izabella Costa, Fernando Dias, Davide Degli Esposti, Mariana Boroni, Zdenko Herceg, Luis Felipe Ribeiro Pinto

**Affiliations:** 1 Molecular Carcinogenesis Program, Brazilian National Cancer Institute, Rua André Cavalcanti, 37–6° Andar, Bairro de Fátima, Rio de Janeiro 20231-050, Brazil; sheila.lima@inca.gov.br (S.C.S.-L.); drdcamuzi@gmail.com (D.C.); pedronicolau.n@gmail.com (P.N.-N.); monique.lopes@inca.gov.br (M.d.S.A.L.); 2 Institute of Head and Neck Studies and Education (InHANSE), Institute of Cancer and Genomic Sciences, University of Birmingham, Birmingham B15 2TT, UK; H.Mehanna@bham.ac.uk (H.M.); n.batis@bham.ac.uk (N.B.); 3 Laboratório de Hanseníase, Instituto Oswaldo Cruz, Fiocruz, Av. Brasil, 4365, Rio de Janeiro 21040-360, Brazil; paulothiago.santos@fiocruz.br; 4 Departamento de Bioquímica, Instituto de Biologia Roberto Alcantara Gomes, Universidade do Estado do Rio de Janeiro, Av. 28 de Setembro 87 fundos, Vila Isabel, Rio de Janeiro 20551-013, Brazil; tatiana.simao@uerj.br; 5 Epigenetics Group, International Agency for Research on Cancer, 150 Cours Albert Thomas, CEDEX 08, 69372 Lyon, France; cuenin@iarc.fr (C.C.); TalukdarF@fellows.iarc.fr (F.R.T.); davide.degli-esposti@inrae.fr (D.D.E.); HercegZ@iarc.fr (Z.H.); 6 Seção de Cirurgia de Cabeça e Pescoço, Instituto Nacional de Câncer—INCA, Praça da Cruz Vermelha, Rio de Janeiro 20230-130, Brazil; izabella.santos@inca.gov.br (I.C.); fdias@inca.gov.br (F.D.); 7 Bioinformatics and Computational Biology Lab, Brazilian National Cancer Institute, Rua André Cavalcanti, 37–1° Andar, Bairro de Fátima, Rio de Janeiro 20231-050, Brazil; mariana.boroni@inca.gov.br

**Keywords:** DNA methylation, head and neck cancer, esophageal squamous cell carcinoma, WNT signaling pathway

## Abstract

**Simple Summary:**

Squamous cell carcinomas of the upper aerodigestive tract are highly incident, lethal, and share the same epithelial lining of origin, risk factors and genetic alterations. However, their biological and clinical behaviors differ, having an impact on patient survival. This study aimed at identifying the main DNA methylation differences between these tumors, giving an overview of the main genomic regions affected, whether DNA methylation gains or losses are more common, the impact on gene expression and the signaling pathways affected. This knowledge will help identifying potential site-specific biomarkers as well as shedding light on whether epigenetic mechanisms explain, at least in part, the diverse behavior of upper aerodigestive tract tumors.

**Abstract:**

Upper aerodigestive tract (UADT) tumors present different biological behavior and prognosis, suggesting specific molecular mechanisms underlying their development. However, they are rarely considered as single entities (particularly head and neck subsites) and share the most common genetic alterations. Therefore, there is a need for a better understanding of the global DNA methylation differences among UADT tumors. We performed a genome-wide DNA methylation analysis of esophageal (ESCC), laryngeal (LSCC), oral (OSCC) and oropharyngeal (OPSCC) squamous cell carcinomas, and their non-tumor counterparts. The unsupervised analysis showed that non-tumor tissues present markedly distinct DNA methylation profiles, while tumors are highly heterogeneous. Hypomethylation was more frequent in LSCC and OPSCC, while ESCC and OSCC presented mostly hypermethylation, with the latter showing a CpG island overrepresentation. Differentially methylated regions affected genes in 127 signaling pathways, with only 3.1% of these being common among different tumor subsites, but with different genes affected. The WNT signaling pathway, known to be dysregulated in different epithelial tumors, is a frequent hit for DNA methylation and gene expression alterations in ESCC and OPSCC, but mostly for genetic alterations in LSCC and OSCC. UADT tumor subsites present differences in genome-wide methylation regarding their profile, intensity, genomic regions and signaling pathways affected.

## 1. Introduction

Upper aerodigestive tract (UADT) tumors are highly incident worldwide and their prognosis vary according to tumor subsite and affected population [1,2]. Oral cavity, laryngeal and oropharyngeal tumors, usually grouped together as Head and Neck (HN) cancer, as well as esophageal neoplasms are highly associated with alcohol and tobacco consumption, and usually develop into a squamous cell carcinoma [3]. Furthermore, a significant proportion of patients affected by these cancers develop a second primary tumor, synchronously or metachronously, either in the same subsite or at related anatomic subsites [4], usually resulting in a worse prognosis [5,6]. These cancers are not metastases, but tumor masses that develop independently of the first primary tumor and carry different driver molecular alterations [7]. Despite these similarities, UADT tumors show distinct clinical behavior according to specific subsites, and the identification of their specific characteristics is important to improve patients’ survival.

Oropharyngeal squamous cell carcinoma (OPSCC) is probably the best example of how tumor segregation according to carcinogenesis mechanisms may impact overall survival. Human Papillomavirus (HPV)-positive cases show better clinical outcome compared with HPV-negative cases, and molecular markers, such as p16, are used for discriminating groups and staging OPSCC [8]. Although not yet applied in clinical practice, recent studies have shown that disrupting mutations in *NSD1* and *NSD2* (Nuclear Receptor Binding SET Domain Proteins 1 and 2) define a group of good prognoses within laryngeal squamous cell carcinoma (LSCC) cases, but not in other HN subsites [9,10]. Therefore, gaining insight into specific molecular alterations present in UADT tumor subsites is of critical importance.

Genome-wide studies have shown that the most common mutational signatures observed in UADT squamous cell carcinomas are those associated with AID/APOBEC (activation-induced cytidine deaminase/apolipoprotein B mRNA editing enzyme, catalytic polypeptide-like) activity [11,12,13], and the most common genetic alteration is *TP53* mutations, leading to the inactivation of this tumor suppressor gene [14,15]. Therefore, genetic alterations are in general shared and might not be sufficient to distinguish these tumor subsites. Conversely, DNA methylation alterations are also involved in tumor initiation and progression [16], and aberrant DNA methylation profiles have been shown to be tissue-specific and less heterogeneous than genetic alterations, underscoring their potential as subsite-specific attractive biomarkers [17]. These characteristics together with the reversibility of epigenetic modifications have resulted in an increasing interest in the field. Nevertheless, few studies have compared global methylation profile of UADT tumor subsites, but did not investigate thoroughly subsite-specific alterations, particularly those affecting signaling pathways disruption such as the WNT pathway [14,18,19]. The WNT pathway plays a central role in development and stemness [20,21,22], and its dysregulation in epithelial tumors is recurrent [23,24,25]. Furthermore, WNT signaling pathway disruption was previously shown to impact on cancer patient prognosis and presents the potential of anti-cancer therapeutic approaches targeting this pathway [20,21,22,25].

The present study aimed to compare UADT squamous cell carcinomas subsite DNA methylome changes, pointing out to their main differences, and to identify potential differences among subsites regarding the WNT pathway.

## 2. Materials and Methods

### 2.1. Patients

In total, 24 esophageal squamous cell carcinoma (ESCC) patients, 21 LSCC patients, 16 oral squamous cell carcinoma (OSCC) patients and 15 OPSCC patients diagnosed at the Brazilian National Cancer Institute (INCA, Rio de Janeiro, Brazil) were included in the study. Additionally, eight OPSCC patients from the PET-Neck trial (Institute of Head and Neck Studies and Education (InHANSE), University of Birmingham) were also included. Esophageal samples were collected as biopsies through endoscopy procedures, with non-tumor adjacent tissue collected 5 cm from the tumor border. HN tumors and adjacent tissue were collected by the Head and Neck Surgical Division from INCA or from Birmingham University Hospital, from patients who had not undergone chemo- or radiotherapy treatment. For oral cavity and laryngeal sites non-tumor tissue was collected from tumor border free margin sites, selected by a pathologist after patient surgery. For oropharyngeal, the non-tumor tissue consisted of samples collected from tonsillectomies of non-cancer patients. All samples were immediately snap-frozen at liquid nitrogen just after collection (INCA), or formalin-fixed and paraffin embedded (FFPE, PET-Neck). Histopathological profiling of all samples was evaluated by the Pathology Department of INCA, and only tumor samples with >70% of tumor cells were included. Patients’ main characteristics are disclosed in Table S1, but briefly the median age varied between 56–63, most were male (ranging from 87.5% in ESCC to 95.7% in OPSCC), tobacco smokers (smoking at least a cigarette a day for at least one year), alcohol drinkers (drinking alcoholic beverages twice a week for at least one year) and most tumors were diagnosed in late stages (III and IV) in all tumor groups. Patient overall survival (OS) varied according to tumor site (Figure S1), with ESCC showing the lowest median OS (9.1 months), followed by LSCC (20.9 months), and OPSCC (22.1 months). For OSCC, it was not possible to calculate de median OS.

All patients signed informed consents for using biological samples as well as clinical and pathology data from patient records. This study was approved by the institutions Ethics Committees and was conducted according to the Declaration of Helsinki.

### 2.2. Methylome Analysis

Genomic DNA was extracted from 16 non-tumor adjacent tissues and 24 tumors from ESCC patients; 12 non-tumor adjacent tissues and 20 tumors from LSCC patients; seven non-tumor adjacent tissues and 15 tumors from OSCC patients; as well as 15 OPSCC samples, all snap-frozen, with DNeasy Blood and Tissue Kit (Qiagen, Hilden, Germany). All non-tumor adjacent tissues were collected from patients who also donated tumor specimens (matched pairs). Genomic DNA was also extracted from FFPE tonsils from nine non-cancer patients, for whom tobacco and alcohol habits information was not available, and FFPE tumor tissue from eight OPSCC patients with QIAamp DNA FFPE Tissue Kit (Qiagen). Sodium bisulfite-treated DNA (EZ DNA Methylation Kit, Zymo Research, Irvine, CA, USA) was used to assess global DNA methylation profiles by microarray using the Infinium Human Methylation 450K BeadChip (Illumina, San Diego, CA, USA), according to manufacturer’s instructions. In the case of FFPE samples, DNA quality was assessed by the Infinium FFPE DNA QC Kit (Illumina, CA, USA) and only samples that passed the established threshold were used in the following experiments. After sodium bisulfite treatment, restoration was performed using the Infinium FFPE DNA Restoration Kit (Illumina, CA, USA). Microarray experiments were performed in three batches, all containing all sample types, both considering frozen and FFPE samples and tumor types. After checking the built-in-controls with GenomeStudio Software (Illumina, CA, USA), Bioconductor packages were used to perform all analyses in R environment. The idat files were used to obtain average beta-values (AVG_betas) with methylumi [26]. AVG_betas represent the ratio between the intensity of the methylated allele and the intensity of all alleles and vary between 0.0–1.0. Cross-reactive probes and polymorphic probes as described by Chen et al. (2013) [27], samples with 1% of probes with a detection *p*-value > 0.05, probes with beadcount <3 in 5% of samples as well as probes having 1% of samples with a detection *p*-value > 0.05 were removed. After applying these filters, one FFPE sample and one adjacent non-tumor samples from a LSCC patient were removed from the analysis (had more than 1% of probes with a detection *p*-value > 0.05). Finally, Singular Value Decomposition was run to estimate the impact of batch effects and ComBat was run to correct for batch effects, both as functions of the Bioconductor package ‘ChAMP’. Furthermore, color bias adjustment (lumi [28]) and probe bias correction with BMIQ method (watermelon [29]) were performed. In all comparisons, moderated t-statistic was used, and *p*-values were adjusted for multiple testing by Benjamini and Hochberg’s method. Probes with an adjusted *p*-value < 0.001 were considered differentially methylated between groups (limma [30]).

DMRs were identified with the methyAnalysis package [31] using the following criteria: false discovery rate (FDR) <0.05, a minimum of 5 probes within the DMR, a maximum gap of 300 bp between two nearby probes to be considered within a same DMR, and DMRs with a gap ≤100 bp were merged.

The 1000 most variable CpG positions were used to calculate the Euclidean distances between samples (separately for non-tumor and tumor samples) that were projected into a 2-d plane using classical multidimensional scaling transformation (minfi [32]).

Genome-wide mean delta beta from each tumor type were used as input to construct heatmaps with the RCircos package.

Over-representation analyses were performed with WEB-based GEne SeT AnaLysis Toolkit [33] using Kyoto Encyclopedia of Genes and Genomes (KEGG) database. For this analysis, genes containing DMRs in each tumor type were used separately and pathways were considered enriched when FDR was less than 0.05.

Information regarding WNT differentially methylated genes by position (promoter and outside promoter) were plotted with PathVisio [34]. When more than one DMR was differentially methylated in a given region, the mean delta beta of all DMRs was represented.

The datasets used during the current study are deposited in the Gene Expression Omnibus (GEO) database, under the accession numbers: GSE178212, GSE178216, GSE178218, GSE178219.

### 2.3. Cellular Component Analysis

R package MethylCIBERSORT (v0.2.0) [35] was used to construct the stromal and immune cells signature matrix. Methylation beta-values from stromal and immune cells built-in the package were used. Methylation beta-values normalization was performed as already mentioned through the BMIQ method. 100 DMPs with median beta-value differences > 0.25 and FDR < 0.01 were considered in the cellular component signature. Deconvolution was performed in CIBERSORTx [36] server with quantile normalization, in relative mode and 1000 permutations.

### 2.4. RNA-Seq Analysis

Gene expression data from ESCC and LSCC cases was retrieved from previous work by the group [23]. Briefly, total RNA was isolated with the RNeasy Kit (Qiagen) and integrity was assessed with RNA 6000 Nano chip (Agilent, Santa Clara, CA, USA). RNA samples with RNA integrity number (RIN) ≥ 8 were used for constructing cDNA libraries (TruSeq RNA, Illumina), which were sequenced in an Illumina HiSeq 2500 platform. Data processing and read mapping were performed as previously described [23]. Differential expression analysis was performed with DESeq2 package [37].

### 2.5. TCGA Data

TCGA gene expression data were retrieved from cBioportal for cancer genomics [38] and DNA methylation data from TCGA Wanderer [39], and used to calculate correlations for selected genes. The DMR beta-value was defined for each sample by calculating the mean of all probes within the DMR. A total of 81 ESCC, 94 LSCC, 233 OSCC and 69 OPSCC samples were included in this analysis.

Information regarding copy number alterations (deletions) and the presence of putative driver mutations in *TAP1*, *AJUBA* and *NOTCH1* for each tumor type was retrieved from cBioportal for cancer genomics [38]. Data from a total of 96 ESCC, 116 LSCC, 248 OSCC and 79 OPSCC samples were included in this analysis.

The datasets analyzed during the current study are available in the cBioPortal for Cancer Genomics repository [cbioportal.org] [Esophageal Carcinoma (TCGA, Firehose Legacy) and Head and Neck Squamous Cell Carcinoma (TCGA, Firehose Legacy)].

## 3. Results

### 3.1. Global DNA Methylation Profile Is Quite Distinct among UADT Squamous Cell Carcinomas

**Figure 1:**
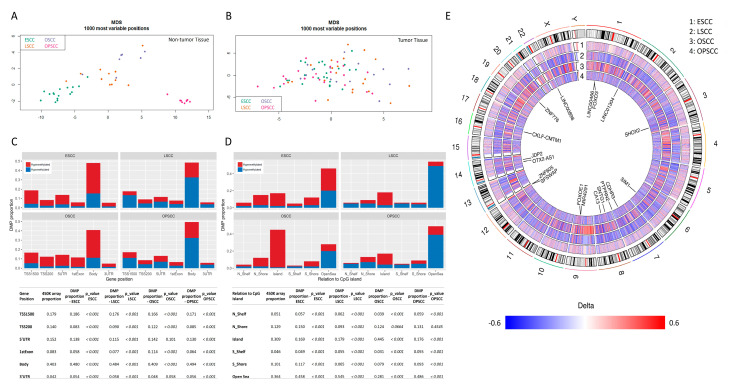
Overall DNA methylation profile of head and neck and esophageal squamous cell carcinomas. (**A**) Principal component analysis of the 1000 most variable CpG sites in non-tumor tissues of esophagus, larynx, oral cavity and oropharynx. (**B**) Principal component analysis of the 1000 most variable CpG sites in squamous cell carcinomas of the esophagus, larynx, oral cavity and oropharynx. (**C**) Bar graphs showing the proportion of hypermethylated and hypomethylated probes, according to their genic location, for each tumor type relative to their non-tumor counterparts. In red: hypermethylated probes; in blue: hypomethylated probes. On the bottom, a table with the proportion of 450 K beadchip probes in each gene region as well as the proportion of DMPs in each gene region by tumor type is shown. *p*-values of chi-squared tests calculated based on the observed and expected absolute numbers of probes are also shown. (**D**) Bar graphs showing the proportion of hypermethylated and hypomethylated probes, according to their relation to CpG islands, for each tumor type relative to their non-tumor counterparts. In red: hypermethylated probes; in blue: hypomethylated probes. On the bottom, a table with the proportion of 450 K beadchip probes according to their relation to CpG island as well as the proportion of DMPs according to their relation to CpG island by tumor type is shown. *p*-values of chi-squared tests calculated based on the observed and expected absolute numbers of probes are also shown. (**E**) Circos plot showing the delta-betas (differences between the mean methylation in tumors and non-tumor tissues) for each probe in the beadchip according to tumor type. From the outer circle to the inner circle: esophageal squamous cell carcinoma (1), laryngeal squamous cell carcinoma (2), oral cavity squamous cell carcinoma (3) and oropharyngeal squamous cell carcinoma (4). The chromosomes are included in the center of the circus plot and genes carrying the 20 DMPs with the largest variances across tumor subsites are shown. Regions in red: hypermethylated sites in tumors relative to their non-tumor counterparts; regions in blue: hypomethylated sites in tumors relative to their non-tumor counterparts, regions in white: probes removed from the analysis for quality reasons or filtering purposes. ESCC, esophageal squamous cell carcinoma; LSCC, laryngeal squamous cell carcinoma; MDS, multidimensional scaling; OSCC, oral cavity squamous cell carcinoma; OPSCC, oropharyngeal squamous cell carcinoma.

The study workflow is shown in Figure S2. Unsupervised analyses revealed differences in the global DNA methylation profile of non-tumor tissue subsites, with oropharynx presenting the most distinct profile (Figure 1A). Among tumors, a more heterogeneous profile was observed, with no clear stratification by subsite in the unsupervised analysis (Figure 1B). This observation led us to perform a stromal and immune cell component analysis by tumor type, based on DNA methylation levels (Figure S3). Fibroblasts and endothelial cells contributed to ~50% of this non-tumor component in most of the samples. The contribution of the immune components was more variable, with ESCC showing a higher percentage of neutrophils, for example.

The supervised comparison revealed a total of 76,210 differentially methylated probes (DMPs) in ESCC, 42,907 in OPSCC, 18,389 in LSCC, and 9978 in OSCC, when compared to their respective non-tumor tissues (*p*-value adjusted by Benjamini and Hochberg’s method—BH < 0.001). Most DMPs were hypermethylated in ESCC and in OSCC (67.6% and 71.83%, respectively), but hypomethylated in LSCC and OPSCC (71.73% and 64.5%, respectively), profile also observed when probes were stratified by gene region (Figure 1C). This stratification revealed that most DMPs encompassed gene bodies in all tumor sites (Figure 1C), as expected by the higher coverage of this region in the beadchip. Nevertheless, they were also more affected than expected by chance (*p* < 0.001) (Figure 1C, Table S2). In ESCC, LSCC and OPSCC, 3′UTR regions were also more frequently affected than by chance (*p* < 0.001) (Figure 1C, Table S2). Promoter regions (TSS200 and TSS1500) were less affected than expected by chance in general. DMPs mapping to the first exon or 5′UTR region of genes were less affected than would be expected by the representation in the beadchip in all tumor subsites except for OSCC (Figure 1C, Table S2).

CpG islands were more commonly hypermethylated in all subsites (Figure 1D), but DMPs mapped to these regions less frequently than expected by chance (*p* < 0.001) in ESCC, LSCC and OPSCC, while in OSCC they were more affected than expected (Table S3, Figure 1D). On the opposite direction, DMPs mapped to open sea regions more frequently than expected in ESCC, LSCC and OPSCC, while in OSCC they were less frequently affected (Table S3, Figure 1D).

Figure 1E shows the genome-wide differential methylation profile for each tumor subsite, evidencing that OSCC shows the highest delta betas (median of –0.28 and 0.29 for hypo and hypermethylated probes, respectively), followed by LSCC (hypo: −0.26 and hyper: 0.27), OPSCC (hypo: −0.21 and hyper: 0.26), and ESCC, which showed the lowest differences in comparison with its non-tumor tissue (hypo: −0.15 and hyper: 0.20) (Figure S4A). Since this could be a consequence of the different number of samples (non-tumor and tumor tissues) used for each tumor subsite, we analyzed this profile only considering the common DMPs, and ESCC indeed showed the lowest median delta betas (Figure S4B). Next, we evaluated whether the mean delta betas could reflect differences within non-tumor tissues or tumors. Figure S4C shows the methylation profile of 1114 common DMPs in these tissues. Taking ESCC as an example, for hypomethylated DMPs, it showed the lowest methylation levels among non-tumor tissues and the highest among tumors, explaining the low delta betas. Similarly, for hypermethylated DMPs, both the non-tumor tissues and tumors’ methylation profiles explain the delta beta differences. ESCC showed the highest methylation levels in NTST and intermediate levels among tumors, again corroborating the low delta betas.

Tumors also showed a different profile of over or underrepresentation of affected chromosomes, with no specific chromosome being commonly affected in the four subsites (Table S4). However, head and neck tumor subsites (LSCC, OSCC and OPSCC) shared an underrepresentation of DNA methylation alterations in chromosomes 9, 17 and 22 and an overrepresentation of chromosome 14.

### 3.2. Differentially Methylated Regions Affect Different Cell Pathways and Different Genes in the Same Cell Signaling Pathways, According to Tumor Subsite

The analysis of differentially methylated regions (DMRs, FDR < 0.05 and CpG sites ≥ 5) corroborated the global methylation profile assessed by DMPs. Hypermethylation was more common in ESCC and OSCC (61.8% of 5833 DMRs and 72.4% of 2145 DMRs, respectively), while LSCC and OPSCC presented mostly hypomethylation (63.2% of 2643 DMRs and 78.5% of 7676 DMRs, respectively). For LSCC and OSCC, the methylation levels of the same DMRs were assessed in TCGA dataset, showing significant differences between tumor and NTST for 98% and 96% of the DMRs, respectively (BH adjusted *p*-value < 0.05). Only 12 out of a total 18,261 DMRs considered (0.066%) were common among the four subsites.

**Figure 2:**
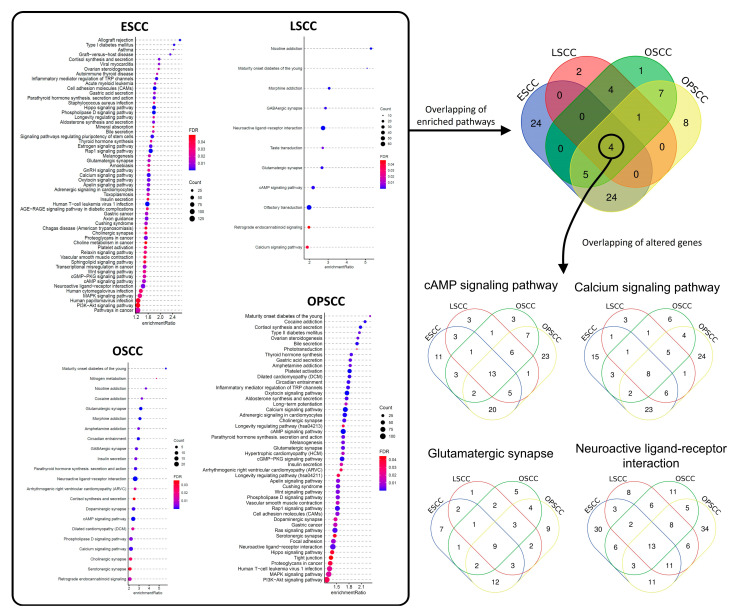
Signaling pathway enrichment analysis with genes affected by differentially methylated regions. DMRs were identified by comparing each tumor type with its corresponding non-tumor tissue. In all comparisons, only DMRs containing at least 5 differentially methylated CpG sites, with FDR < 0.05 were considered. Genes with at least one DMR were included in the Over-representation analysis (ORA) using KEGG database to identify enriched signaling pathways. In the highlighted rectangle in the Figure, enriched pathways for each tumor type are shown (FDR < 0.05). The overlapping of enriched pathways (color filled Venn diagram) showed that 4 signaling pathways were over-represented in all tumor types, cAMP Signaling Pathway, Calcium Signaling Pathway, Glutamatergic Synapse, and Neuroactive Ligand-Receptor Interaction. However, the overlapping of DMR-carrying genes showed that (non-colored filled Venn diagrams) the genes affected varied in the different tumor types. ESCC, esophageal squamous cell carcinoma; LSCC, laryngeal squamous cell carcinoma; OSCC, oral cavity squamous cell carcinoma; OPSCC, oropharyngeal squamous cell carcinoma.

Next, over-representation analysis (ORA) was performed to identify the KEGG (Kyoto Encyclopedia of Genes and Genomes) signaling pathways enriched for genes affected by DMRs in each tumor subsite (FDR < 0.05, Table S5). Figure 2 shows that ESCC presented the highest number of affected pathways (57), followed by OPSCC (49), OSCC (22), and LSCC (11). The overlap of enriched pathways evidenced that only four out of 127 affect pathways (3.1%) were common among the four subsites. These were cAMP Signaling Pathway, Calcium Signaling Pathway, Glutamatergic Synapse, and Neuroactive Ligand-Receptor Interaction (Figure 2). Nevertheless, a proportion of the genes affected in these four pathways were different among the subsites (Figure 2, Table S5).

### 3.3. Methylation Levels of DMRs in WNT Signaling Genes Are Potentially Associated with Gene Expression

WNT signaling pathway was found enriched by genes carrying DMRs in ESCC and OPSCC, but not in LSCC and OSCC. Abnormal activation of this pathway has been constantly associated with epithelial tumor development and prognosis [22,23,24,25]. Therefore, we performed a deeper analysis of WNT-associated genes in these tumors.

**Figure 3:**
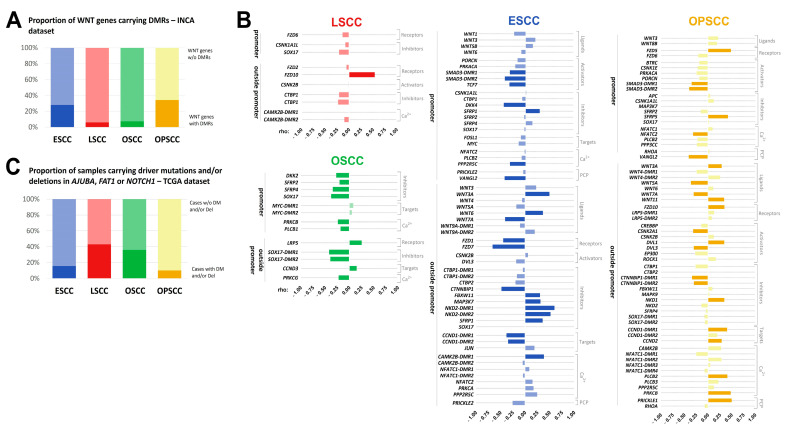
The molecular mechanisms affecting the WNT signaling pathway are different in ESCC, LSCC, OSCC and OPSCC. (**A**) Bar graph showing the percentage of genes of the WNT signaling pathway carrying at least one DMR in each tumor type within our dataset. ESCC, blue; LSCC, pink; OSCC, green; OPSCC, yellow. Dark and light colors represent the frequency of altered and non-altered genes, respectively. (**B**) Bar graphs showing the correlation between the methylation levels of each affected WNT DMR and the mRNA expression of its associated gene in each tumor type. For this analysis, TCGA datasets were used. Spearman’s correlation rho is shown in X-axis. Genes were grouped according to their function in WNT signaling pathway and DMRs were grouped according to their location, within promoters or outside promoters. ESCC, blue; LSCC, pink; OSCC, green; OPSCC, yellow. Dark and light colors represent the significant (BH adjusted *p*-value < 0.05) and not significant correlations, respectively. (**C**) Bar graph showing the percentage of cases carrying driver mutations (DM) and/or deep deletions in *AJUBA*, *FAT1* and *NOTCH1* in each tumor type within TCGA dataset. ESCC, blue; LSCC, pink; OSCC, green; OPSCC, yellow. Dark and light colors represent the frequency of altered and non-altered genes, respectively. ESCC, esophageal squamous cell carcinoma; LSCC, laryngeal squamous cell carcinoma; NTST, non-tumor surrounding tissue; NTT, non-tumor tissue; OSCC, oral cavity squamous cell carcinoma; OPSCC, oropharyngeal squamous cell carcinoma.

**Figure 4:**
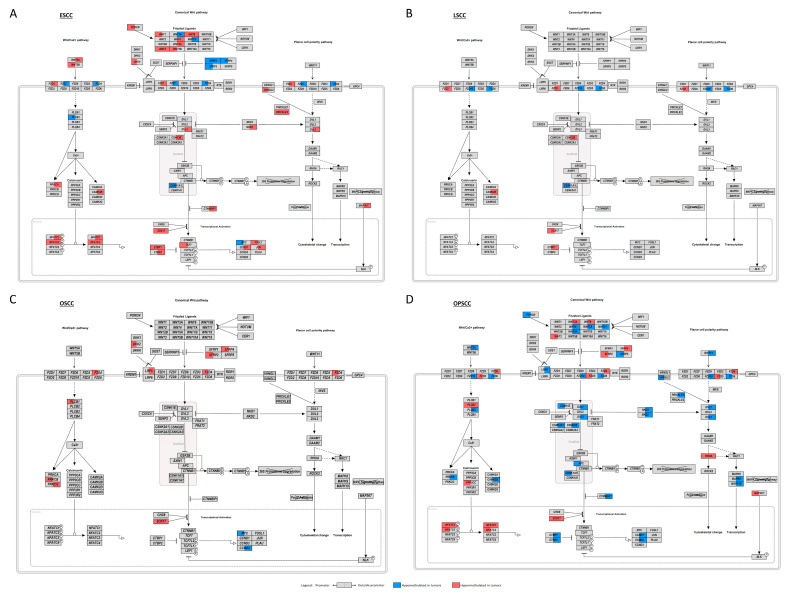
WNT pathway disruption by DNA methylation alterations in ESCC, LSCC, OSCC and OPSCC. Schematic representation of the WNT pathway showing the genes involved and their DMR alterations in: (**A**) esophageal squamous cell carcinoma (ESCC); (**B**) laryngeal squamous cell carcinoma (LSCC); (**C**) oral cavity squamous cell carcinoma (OSCC); (**D**) oropharyngeal squamous cell carcinoma (OPSCC). Each rectangle represents a gene involved in the pathway and is divided into two halves: the left halves represent the DMRs annotated to promoters; and the right halves represent the DMRs annotated outside promoters. The colors indicate whether the region was found to be hypermethylated (red) or hypomethylated (blue) in each tumor relative to their respective non-tumor tissue. When more than one DMR was differentially methylated in a given region, the mean delta beta of all DMRs was represented.

From the 150 genes included in the WNT signaling pathway map from KEGG database, 51 (34%, with 64 DMRs) were affected by DMRs in OPSCC, 42 (28%, with 57 DMRs) in ESCC, but only 11 (7.3%, with 14 DMRs) in OSCC, and 9 (6%, with 12 DMRs) in LSCC (Figure 3A, Table S6). DMRs in genes involved in the WNT pathway were classified according to their location (promoter and outside promoter) and a representation of the pathway containing this information was designed (Figure 4). To understand whether these DNA methylation alterations would have a functional impact, we assessed gene expression in ESCC and LSCC in our dataset, representing tumor subsites with a high and low number of affected genes, respectively. In ESCC, 24 out of 42 genes carrying DMRs (57.1%) were differentially expressed (Figure S5). In LSCC, only three out of nine DMR-affected genes showed differential expression (30%), (Figure S6).

To validate the potential impact of DMRs identified here on gene expression, we used the TCGA datasets for each tumor subsite included in the present study. In this analysis, the correlation between the mean methylation value of all CpG sites included in the DMR and the mRNA expression of the respective gene was assessed. WNT signaling genes carrying more than one DMR in a specific tumor subsite had the correlation calculated separately for each DMR. The methylation levels of at least one DMR was significantly correlated with mRNA expression (BH adjusted *p*-value < 0.05) in 81.8% of the genes in OSCC, 40.5% in ESCC, 39.2% in OPSCC, and only 12.5% in LSCC (Figure 3B, Table S6).

Although a relatively low number of genes was affected by DMRs in OSCC, most of these DMRs were significantly correlated with expression in TCGA dataset (Figure 3B). The pathway inhibitors *DKK2*, *SFRP2*, *SFRP4* and *SOX17* showed hypermethylated DMRs in the promoter region that were inversely correlated with mRNA expression (Figure 3B, Table S6), suggesting their downregulation in *OSCC*. SOX17 also carried a hypermethylated DMR outside the promoter region inversely correlated with expression.

In ESCC, only DMRs outside promoters were significantly correlated with the expression of WNT ligands (Figure 3B). Of note, *WNT7A* was found hypomethylated and overexpressed in our dataset, and TCGA data evidenced an inverse correlation between methylation and expression (Figure 3B, Table S6). Considering WNT receptors, *FZD7* showed a hypomethylated DMR outside the promoter region and mRNA overexpression in ESCC respective to NTST in our dataset, and its methylation levels were inversely correlated with expression in TCGA. The downregulation and hypermethylation (outside promoter) of *CTNNBIP1*, a pathway inhibitor, was detected in our dataset, followed by an inverse correlation between DMR methylation and mRNA expression in TCGA (Figure 3B, Table S6).

OPSCC showed DMRs both in the promoters and outside the promoter region of WNT signaling genes. However, the latter were more commonly correlated with gene expression (Figure 3B). Regarding WNT ligands, DMRs in *WNT5A* and *WNT7A* were hypomethylated and inversely correlated with gene expression (Figure 3B, Table S6). WNT3A was hypermethylated and its DMR methylation levels were positively correlated with expression. In all these cases, an overexpression of the ligands would be expected to take place. Conversely, *WNT11* DMR was hypomethylated and positively correlated with expression, suggesting its downregulation.

In LSCC, the methylation levels of only one DMR located outside the promoter region of *FZD10* was significantly and positively correlated with mRNA expression in TCGA dataset (Figure 3B). Since this DMR was hypomethylated in tumors in our dataset (Table S6), a gene upregulation would be expected. However, our RNA-seq data showed no significant differences (Figure S6).

### 3.4. WNT Signaling Pathway May Be Affected by Different Molecular Mechanisms in UADT Subsites

WNT signaling activation in HN tumor subsites has been associated with genetic alterations in *AJUBA*, *FAT1* and *NOTCH1* [24]. We evaluated whether the frequency of genetic alterations would be higher in the subsites that presented a lower proportion of WNT genes affected by DMRs. With this purpose, we evaluated the proportion of cases of each tumor subsite carrying potential driver mutations and/or deep deletions in *AJUBA*, *FAT1* and *NOTCH1* in TCGA datasets. The frequency of genetic alterations in either of the three genes was 43.1% in LSCC, 35.9% in OSCC, 15.6% in ESCC, and 10.1% in OPSCC (Figure 3C).

Finally, we checked whether the WNT signaling pathway was activated in ESCC, LSCC and OSCC, although the associated molecular mechanisms seem to differ. From a list of 49 direct transcriptional targets of the pathway in mammals (the WNT homepage [40]), 32 (65.3%) were differentially expressed (BH adjusted *p*-value < 0.05) in ESCC (being 28 up- and 4 downregulated), 29 (59.2%) in OSCC (being 22 up- and 7 downregulated) and 23 (46.9%) in LSCC (being 22 up- and 1 downregulated) (Figure S7), suggesting an activation of the pathway in the evaluated tumor subsites.

## 4. Discussion

There is limited information regarding the comprehension of the specific epigenetic alterations involved in UADT tumor subsites [41,42,43,44]. In this study we showed that four subsites of UADT tumors analyzed in this study, ESCC, OSCC, OPSCC and LSCC, present marked differences among them regarding general profile, intensity, and genomic region of methylation changes, resulting in different genes and pathways affected, exemplified by the WNT pathway alteration analysis in the four subsites.

We showed that the general DNA methylation profile differs among the non-tumor epithelium of the different UADT subsites, evidencing that, although they are lined by a common squamous mucosa, tissue-specific phenotypes can be noticed even before neoplastic transformation. However, we cannot rule out that at least some of the observed differences can be a consequence of different sample source. For OPSCC, opposite to the other tumor subsites, for which fresh-frozen NTST was collected, the non-tumor tissue consisted of FFPE tonsils from individuals going through uvulopalatopharyngoplasty for conditions not associated with cancer. Tonsils were used based on previous studies and on the high prevalence of OPSCC arising from these organs [45,46,47,48,49,50,51], but future studies on OPSCC NTST should be performed to confirm our findings.

When considering all DMPs, ESCC and OSCC presented a clear hypermethylation, whereas LSCC and OPSCC showed a hypomethylation profile. Poage et al. (2011) [41] compared the global methylation profile of HN tumors and showed that LINE-1 and 27k Illumina Infinium Methylation Beadchip median methylation levels were higher in oral, followed by pharynx and larynx squamous cell carcinomas. In a multivariable regression, AluYB8 methylation levels were also lower in LSCC relative to OSCC [41]. These data are in accordance with our findings, but the authors directly compared tumors, while we compared the differences relative to non-tumor counterparts. In a similar experimental design to ours, LSCC and OSCC were shown to be mostly hypomethylated, while OPSCC was mostly hypermethylated relative to normal adjacent tissue [42]. Apart from LSCC, our results show the opposite profile, what could be associated with the different platforms used in the two studies, 27 K by Lleras and colleagues and 450 K by us. Nevertheless, from the 25 genes carrying hypermethylated DMPs in all tumor subsites in the cited study, 17 (68%) were also commonly hypermethylated in our dataset, including ESCC.

TCGA network has proposed a classification for HNSCC based on DNA methylation, in which tumors could be divided in hypermethylated, hypomethylated, normal-like and CpG island methylated [14]. More specifically, the hypomethylated subgroup was enriched for *NSD1*-mutated tumors. Since *NSD1* mutations have been shown to be enriched in LSCC [52], this genetic alteration could contribute to the establishment of a hypomethylated phenotype in these tumors [14]. Methylation profiles have also been used before to evaluate and subdivide tumors from the same histology within TCGA datasets [43]. Squamous cell carcinomas, including lung, cervical, bladder, head and neck and esophagus, were classified in 5 clusters, based on the methylation profile of 905 differentially methylated and differentially expressed genes in at least one tumor type. Cluster 4, hypermethylated, and cluster 5, hypomethylated, were mostly composed by HNSCC. In cluster 5, a higher proportion of *NSD1* mutations was found, what could explain the hypomethylated profile, and suggests a higher representation of LSCC. The hypermethylation profile observed in cluster 4 was associated with *CASP8* and *HRAS* mutations. A higher frequency of these mutations has been described before in OSCC relative to other HNSCC subsites [52]. This suggests that cluster 4 might be enriched for OSCC, corroborating the hypermethylation profile described here.

OSCC clearly presented a unique hypermethylation profile, particularly enriched in CpG islands. A CpG island methylator phenotype (CIMP) was previously reported in HNSCC and was indeed more frequent among OSCC patients [53]. However, this group of patients, called CIMP-Atypical, was enriched for females and never or former smokers (>15 years without smoking), differing from our dataset. The patients included in our study reflect the general OSCC patient profile, being mostly males (93.3%) and current smokers (>80%) [54]. Furthermore, OSCC patient profile was not different from patients with tumors at other subsites in our study. Therefore, etiology might not be the cause of CIMP. Brennan K. and colleagues also associated the hypermethylation phenotype with mutations in *CASP8*, *HRAS* and *NOTCH1* [53], the latter being more commonly, but not exclusively, mutated in OSCC in our analyses.

Promoter hypermethylation of 11 tumor suppressor genes has been previously correlated with EZH2 expression in HNSCC [55]. This histone methyltransferase is part of the Polycomb complex and catalyzes H3K27 trimethylation, a mark of gene silencing [56]. The association of *EZH2* overexpression with both gene-specific and global hypermethylation has been described before for prostate cancer, small cell lung carcinoma and cell sarcoma of the kidney, suggesting the role of this histone modifier in directing hypermethylation [57,58,59]. Although such association has not been described in OSCC, mostly hypermethylated according to our results, *EZH2* higher expression was shown to be an independent factor for OSCC development within oral leukoplakia patients [60] and associated with radioresistance [61]. Interestingly, global hypermethylation has been observed in radioresistant HNSCC before [62]. ESCC, the other hypermethylated UADT subsite described here, also presents *EZH2* overexpressed [63] and associated with a poor prognosis [64]. These data suggest a role for *EZH2* in OSCC and ESCC and, although speculative, this might be associated, at least in part with the hypermethylation profile observed here, and future studies should test this hypothesis.

Another surprising difference among tumor subsites evaluated was the variable median delta betas, both considering hypo and hyper DMPs, with ESCC showing the lowest differences. This could indicate that esophageal mucosa shows the most distinct methylation profile when considering the other UADT linings. This may be a consequence of inherent molecular characteristics of the esophageal squamous epithelium but is also clearly caused by subsite specific neoplastic transformation. The methylation profile of normal tissues from cancer patients have been shown to differ and this might be associated with cancer development [65].

We observed a difference in DMRs similar to that seen for DMPs. Genes affected by DMRs resulted in a total of 127 pathways potentially altered, but only 3.1% (four) of these were common to all subsites. Even among the low proportion of similar target pathways, the genes affected were in most cases different. These data indicate that epigenetic alterations affect distinct pathways and genes among different UADT tumor subsites. One of the differentially affected pathways was the WNT signaling pathway. The WNT signaling pathway activation has been recurrently reported in UADT tumors from preneoplastic lesions to more advanced tumors, being associated with patients’ outcome [66,67,68,69,70,71,72,73,74,75]. This pathway is crucial for the development of the epithelial linings, but in adult epithelial cells it becomes silent, and its reactivation seems to play a driver role in neoplastic transformation [71,73,74]. Studies using in vitro and in vivo models have shown that tobacco-mimicking compounds and HPV infection can induce the activation of WNT pathway [76,77,78,79]. However, the molecular mechanisms that lead to its activation are not so clear. Genetic alterations in WNT-activating genes are rare in UADT tumors, while inactivating mutations in non-canonical genes (*FAT1*, *AJUBA* and *NOTCH1*) might be associated with the disruption of the WNT pathway in a subset of UADTs, leading to beta-catenin stabilization [24].

However, here we show that not only the molecular mechanisms vary depending on subsite, but also the genes and the affected regulatory regions can differ. LSCC and OSCC showed a higher frequency of disrupting genetic alterations in the so-called non-canonical WNT genes (43.1% and 35.9%, respectively), *AJUBA*, *FAT1* or *NOTCH1*, and a relatively small number of WNT genes affected by DMRs. LSCC had only one gene (*FZD10*) with a correlation between methylation and expression, suggesting a small contribution of DNA methylation dysregulation of WNT genes to pathway disruption. OSCC also showed a relatively small number of WNT genes affected by DMRs, but their methylation levels were significantly correlated with mRNA expression in most cases. This profile was mainly observed for WNT pathway inhibitors and corroborated previous studies that showed *SFRP2*, *SFRP4*, *SFRP5*, *WIF1*, *DKK3* and *DAB2* (all WNT antagonists) promoter hypermethylation [69,80,81]. In addition, for *SOX17*, recently shown to be epigenetically silenced in OSCC, with its hypermethylation associated with a poor prognosis [66], we detected DMRs both in the promoter and outside the promoter region, both associated with gene expression. This suggests the contribution of both epigenetic and genetic alterations activating WNT pathway in OSCC.

Genetic alterations in the non-canonical WNT genes are present in only 15.6% of ESCC and 10.1% of OPSCC, and epigenetic mechanisms seem to be at least one of the causes of WNT signaling disruption in these tumor subsites. Here we identified DMRs in all classes of WNT genes, including ligands, receptors, activators, inhibitors, targets, and genes involved in WNT/Ca^2+^ signaling and planar cell polarity (PCP) pathway. While few studies have shown such association for OPSCC before [69,82,83], most data available for ESCC point to a role of aberrant DNA methylation in the silencing of WNT negative regulators [84,85,86,87,88]. Therefore, our study suggests a broader range of genes affected by this epigenetic mechanism that may go beyond promoters.

The data presented here show a quite complex tumor specific subsite methylome landscape. Although we applied stringent filters and quality control approaches, the different tissue source for OPSCC (fresh-frozen and FFPE) was a limitation of the present work and should be addressed in future studies. Nevertheless, overall DNA methylation profile in tumors was highly heterogeneous, corroborating previous studies [13,38,39]. This might be associated both with tumor cell epigenetic clonality and variations of the stromal and immune cell component, as shown in Figure S3 and previously assessed by other authors [89,90]. Indeed, the immune infiltrate, usually assessed by gene expression levels, was already shown to vary among ESCC and HNSCC patients [91,92,93] and might explain, at least in part, tumor heterogeneity in terms of DNA methylation profile. The number of samples analyzed was limited and we were not able to evaluate the impact of etiological factors on the DNA methylation profiles identified due to the quite homogeneous characteristics of patients, mostly heavy smokers, heavy drinkers and HPV-negative. However, it is important to mention that this is the actual profile of UADT patients in general [51,54,94,95]. Therefore, the molecular heterogeneity within these tumor types might be even more pronounced. Defining these profiles may indicate new potential diagnostic and prognostic biomarkers, and point to new therapeutic routes for tumors with very poor outcomes.

## 5. Conclusions

The present study gives a comprehensive overview of the differences in the DNA methylation profile of ESCC, LSCC, OSCC and OPSCC, showing that these UADT subsite tumors present differences in profile, intensity and regions affected by methylation alterations. The WNT signaling pathway, constantly activated in epithelial tumors, was differentially affected among UADT subsites, presenting methylation alterations particularly in ESCC and OPSCC, whereas genetic alterations capable of activating it are more pronounced in LSCC and OSCC.

## Data Availability

The datasets used during the current study are deposited in the Gene Expression Omnibus (GEO) database, under the accession numbers: GSE178212, GSE178216, GSE178218, GSE178219.

## Supplementary Materials

The following are available online at https://www.mdpi.com/article/10.3390/cancers13123014/s1, Table S1: Characteristics of the patients included in the study, Figure S1: Patient overall survival according to tumor site, Figure S2: Study workflow, Figure S3: Stromal and immune cell contribution in ESCC and HNSCC, Table S2: Differentially methylated probes stratified by their gene position in ESCC, LSCC, OSCC and OPSCC, Table S3: Differentially methylated probes stratified by their relation to CpG island in ESCC, LSCC, OSCC and OPSCC, Figure S4: DMP delta beta differences reflect non-tumor and tumor differences, Table S4: Differentially methylated probes stratified by chromosomes in ESCC, LSCC, OSCC and OPSCC, Table S5: Over-representation (ORA) analysis of genes carrying DMRs in ESCC, LSCC, OSCC and OPSCC, Table S6: DMR description and correlation with the expression of the associated gene in ESCC, LSCC, OSCC and OPSCC, Figure S5: Gene expression profile of WNT genes carrying DMRs in ESCC, Figure S6: Gene expression profile of WNT genes carrying DMRs in LSCC, Figure S7: WNT target genes are upregulated in ESCC, LSCC and OSCC.
