# Supplementary material for: Upper Aerodigestive Tract Squamous Cell Carcinomas Show Distinct Overall DNA Methylation Profiles and Different Molecular Mechanisms behind WNT Signaling Disruption
Source: Cancers (Basel). 2021 Jun 16;13(12):3014. doi: 10.3390/cancers13123014 (PMC8234055; doi:10.3390/cancers13123014)
Supplement: Supplementary file 1 [file cancers-13-03014-s001.zip › Supplementary Materials_post-revision.pdf]

Supplementary Materials

Table S1: Characteristics of the patients included in the study.

Figure S1: Patient overall survival according to tumor site. Kaplan-Meier survival curve according to tumor site, with follow-up duration of five years after diagnosis. Censored values (+) indicate the last known follow-up time for the corresponding individuals. The numbers indicate the median overall survival for each tumor site. ESCC, esophageal squamous cell carcinoma; LSCC, laryngeal squamous cell carcinoma; ND, not determined, OSCC, oral squamous cell carcinoma; OPSCC, oropharyngeal squamous cell carcinoma.

Figure S2: Study workflow.

Figure S3: Stromal and immune cell contribution in ESCC and HNSCC. Methylation beta-values from stromal and immune cells built-in the R package MethylCIBERSORT (v0.2.0) were used to determine the relative abundance of non-tumor cells within tumor samples. Each graph represents a tumor type, and each column represents a tumor sample. ESCC, esophageal squamous cell carcinoma; LSCC, laryngeal squamous cell carcinoma; OSCC, oral squamous cell carcinoma; OPSCC, oropharyngeal squamous cell carcinoma.

Table S2: Differentially methylated probes stratified by their gene position in ESCC, LSCC, OSCC and OPSCC.

Table S3: Differentially methylated probes stratified by their relation to CpG island in ESCC, LSCC, OSCC and OPSCC.

Figure S4: DMP delta beta differences reflect non-tumor and tumor differences. (A) Boxplot showing the mean delta betas of all hypo- and hypermethylated DMPs in each tumor subsite. (B) Boxplot showing the mean delta betas of all hypo- and hypermethylated common DMPs by tumor subsite. (C) Heatmap showing the methylation profile of all common DMPs among tumor

subsites in non-tumor and tumor tissues. Blue and red represent lower and higher methylation levels, respectively.

Table S4: Differentially methylated probes stratified by chromosomes in ESCC, LSCC, OSCC and OPSCC.

Table S5: Over-representation (ORA) analysis of genes carrying DMRs in ESCC, LSCC, OSCC and OPSCC.

Table S6: DMR description and correlation with the expression of the associated gene in ESCC, LSCC, OSCC and OPSCC.

Figure S5: Gene expression profile of WNT genes carrying DMRs in ESCC. Box plots showing the expression levels of each gene involved in the WNT signaling pathway for which at least one DMR was detected in ESCC relative to non-tumor surrounding tissue. Genes are organized according to their function. Ligands: *WNT1, WNT3A, WNT4, WNT5A, WNT5B, WNT6, WNT7A, WNT9A*. Receptors: *FZD1, FZD7*. Activators: *CSNK2B, PORCN, PRKACA, SMAD3, TCF7, DVL3*. Inhibitors: *CTBP1, CTBP2, CTNNBIP1, DKK4, FBXW11, MAP3K7, NKD2, SFRP1, SFRP2, SFRP5, SOX17*. Targets: *CCND1, FOSL1, JUN, MYC*. WNT/Ca^{2+} signaling: *CAMK2B, NFATC1, NFATC2, PLCB2, PPP2R5C, PRKCA*. Planar cell polarity pathway: *PRICKLE2, ROCK1, VANGL2*.

Figure S6: Gene expression profile of WNT genes carrying DMRs in LSCC. Box plots showing the expression levels of each gene involved in the WNT signaling pathway for which at least one DMR was detected in LSCC relative to non-tumor surrounding tissue. Genes are organized according to their function. Receptors: *FZD2, FZD6, FZD10*. Activators: *CSNK2B*. Inhibitors: *CSNK1A1L, CTBP1, SOX17*. WNT/Ca^{2+} signaling: *CAMK2B*. Planar cell polarity pathway: *ROCK1*.

Figure S7: WNT target genes are upregulated in ESCC, LSCC and OSCC. Heatmaps showing the expression profile of significantly altered (BH adjusted p-value < 0.05) WNT target genes in esophageal (ESCC), laryngeal (LSCC) and oral cavity squamous cell carcinomas (OSCC) and

corresponding non-tumor surrounding tissues (NTST). On the right side of the heatmaps de mean log2 fold-change between tumors and NTST (T_log2FC) is represented in pink for upregulated and in blue for downregulated genes.
