# Supplementary figures and images for: Upper Aerodigestive Tract Squamous Cell Carcinomas Show Distinct Overall DNA Methylation Profiles and Different Molecular Mechanisms behind WNT Signaling Disruption
Source: Cancers (Basel). 2021 Jun 16;13(12):3014. doi: 10.3390/cancers13123014 (PMC8234055; doi:10.3390/cancers13123014)

# Overall survival - INCA dataset

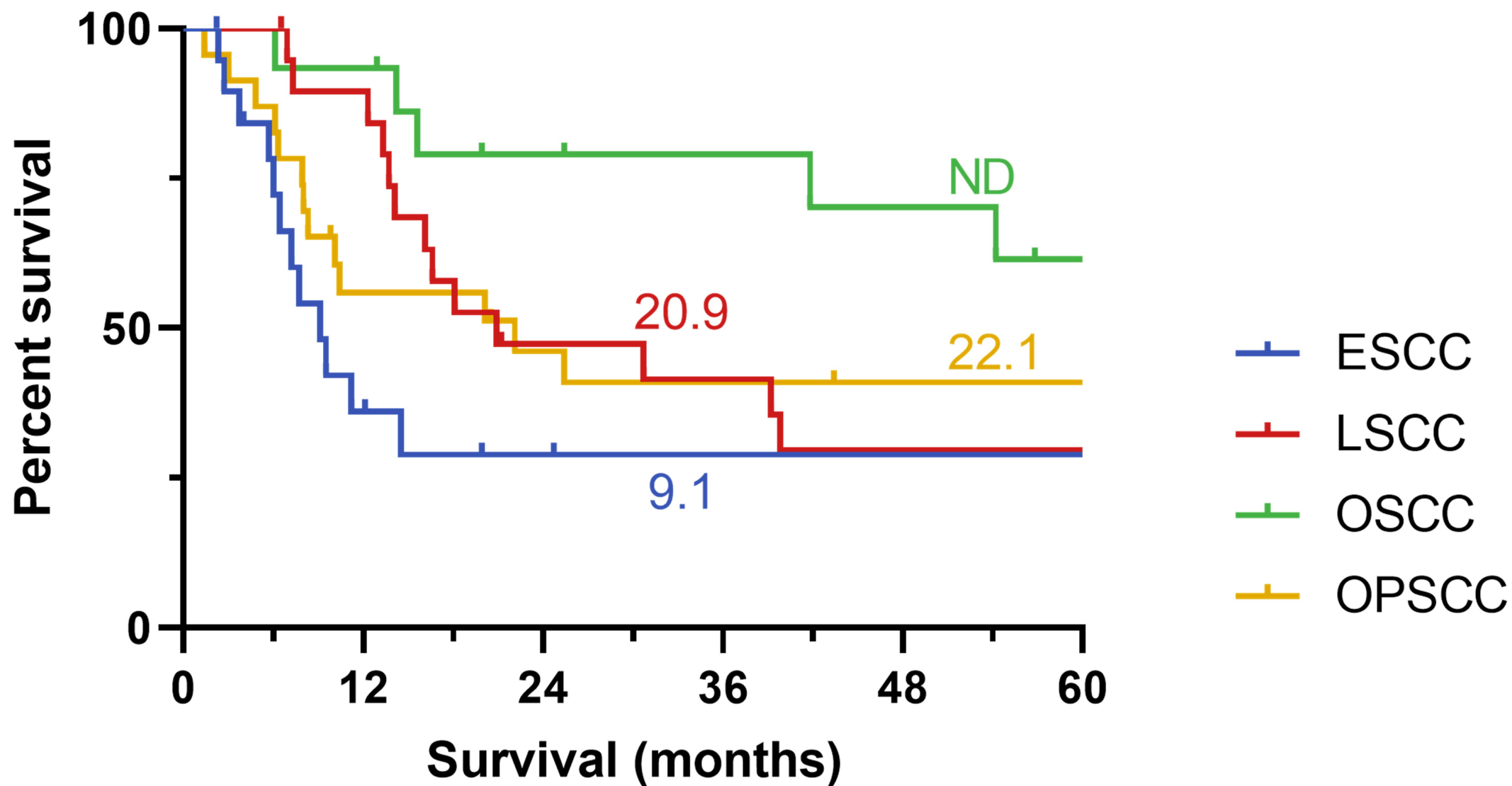

Log-rank p-value = 0.0048

Supplement: Supplementary file 1 [file cancers-13-03014-s001.zip › Figure S1.pdf]

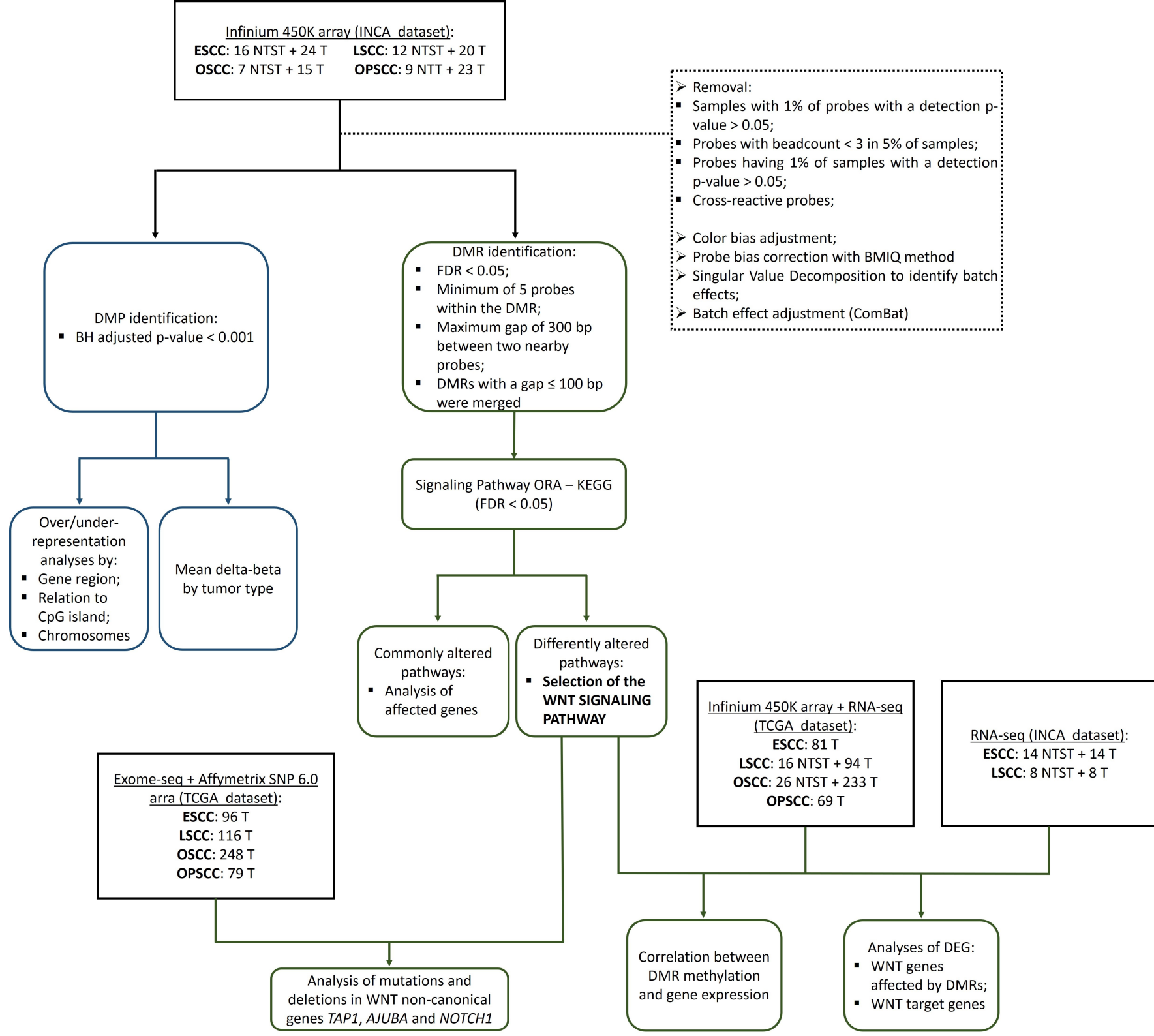

Supplement: Supplementary file 1 [file cancers-13-03014-s001.zip › Figure S2.pdf]

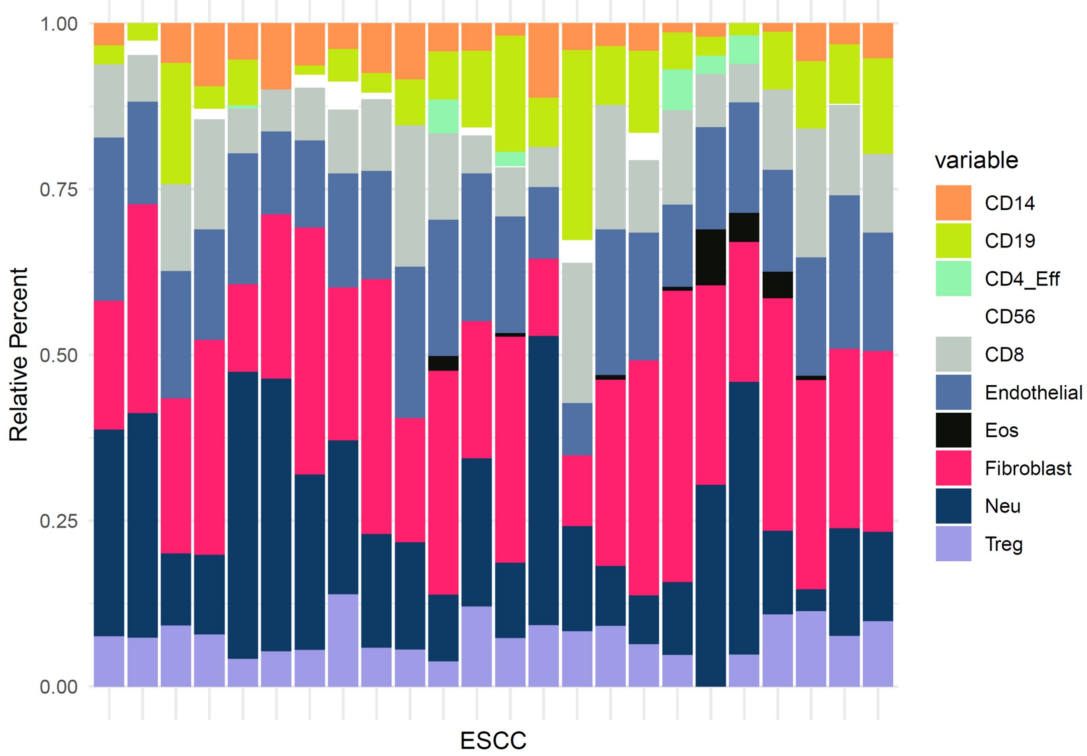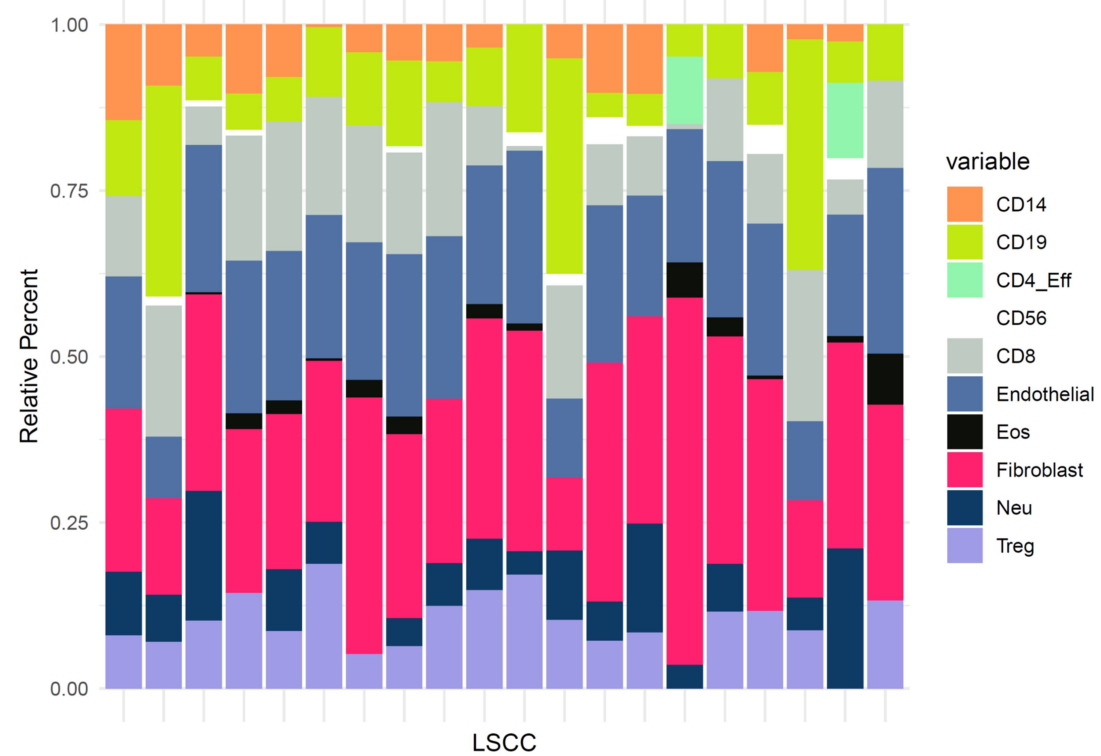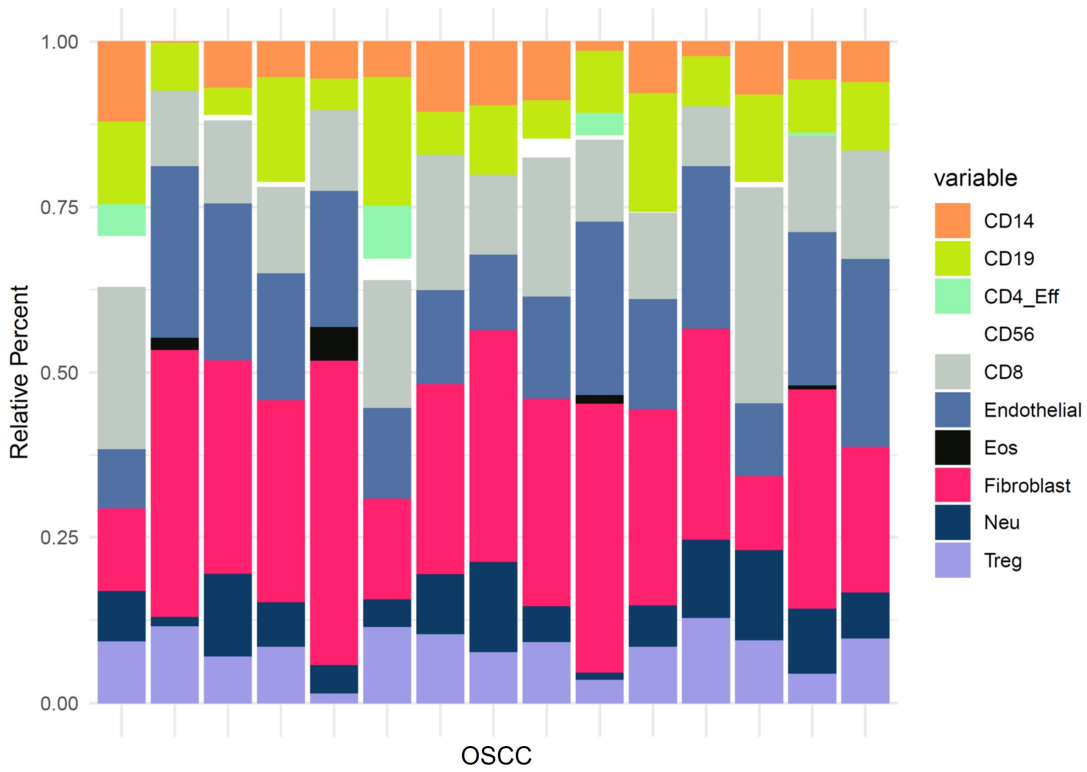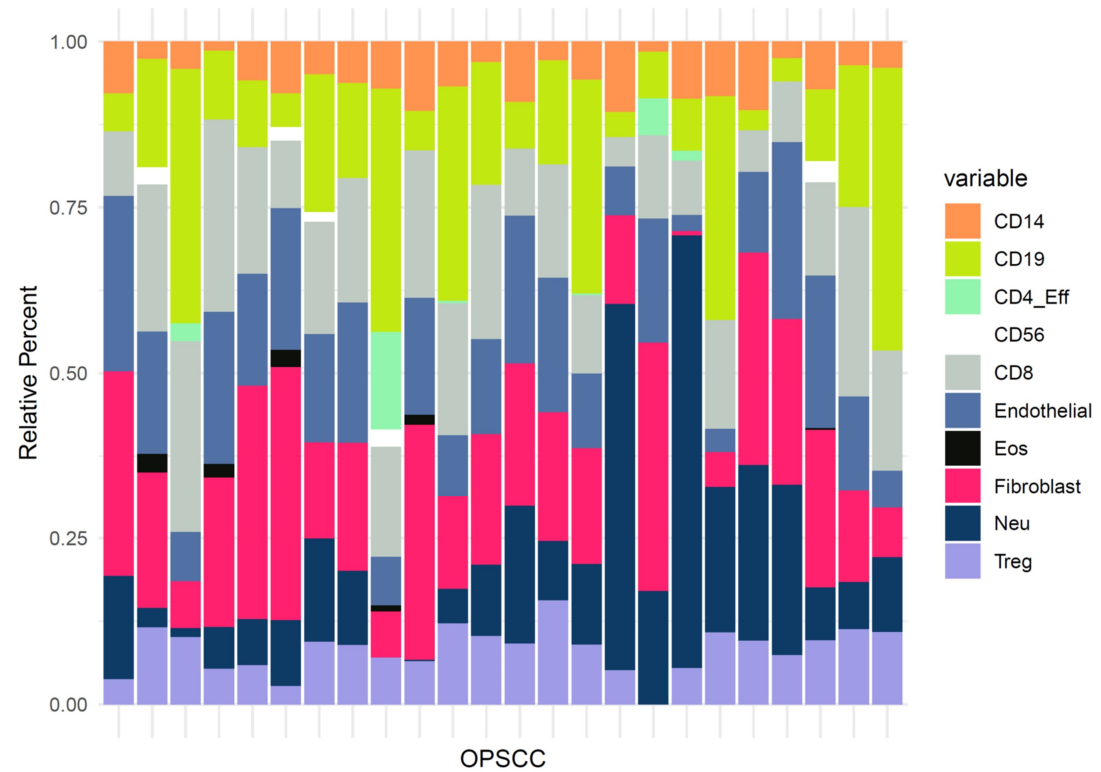

Supplement: Supplementary file 1 [file cancers-13-03014-s001.zip › Figure S3.pdf]

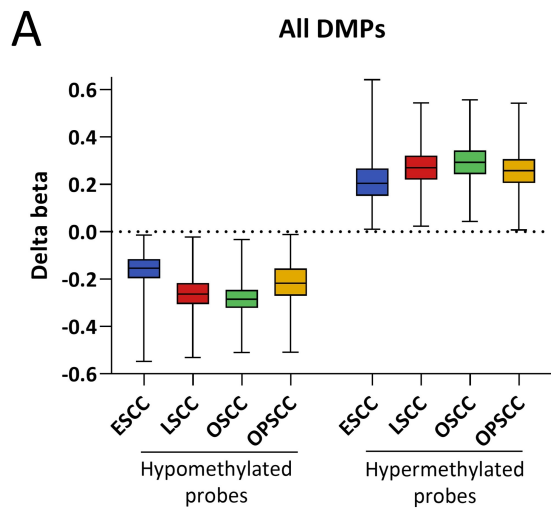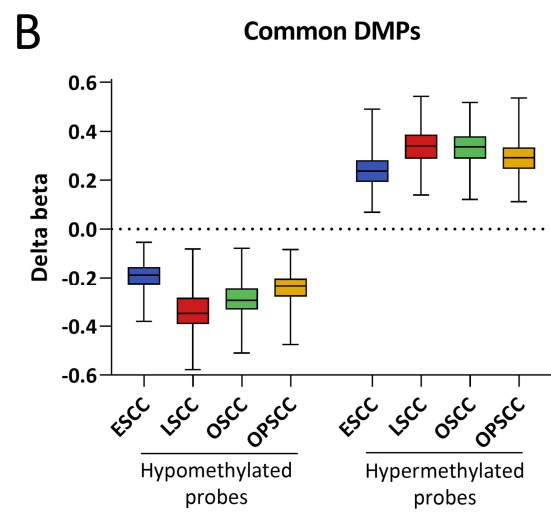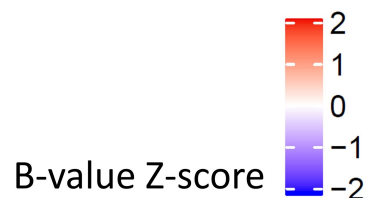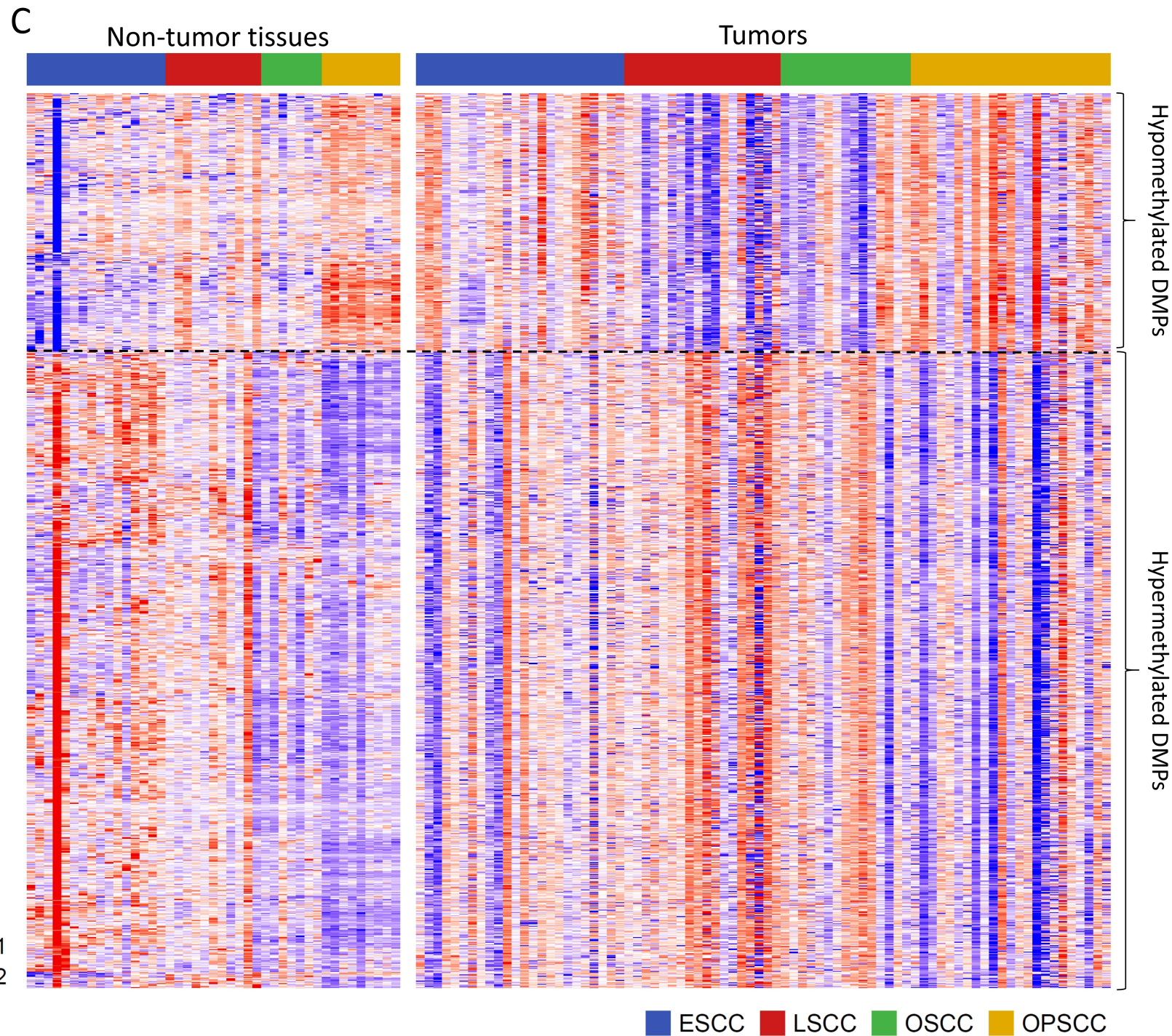

Supplement: Supplementary file 1 [file cancers-13-03014-s001.zip › Figure S4.pdf]

# WNT signaling pathway

Tissue NTST ESCC

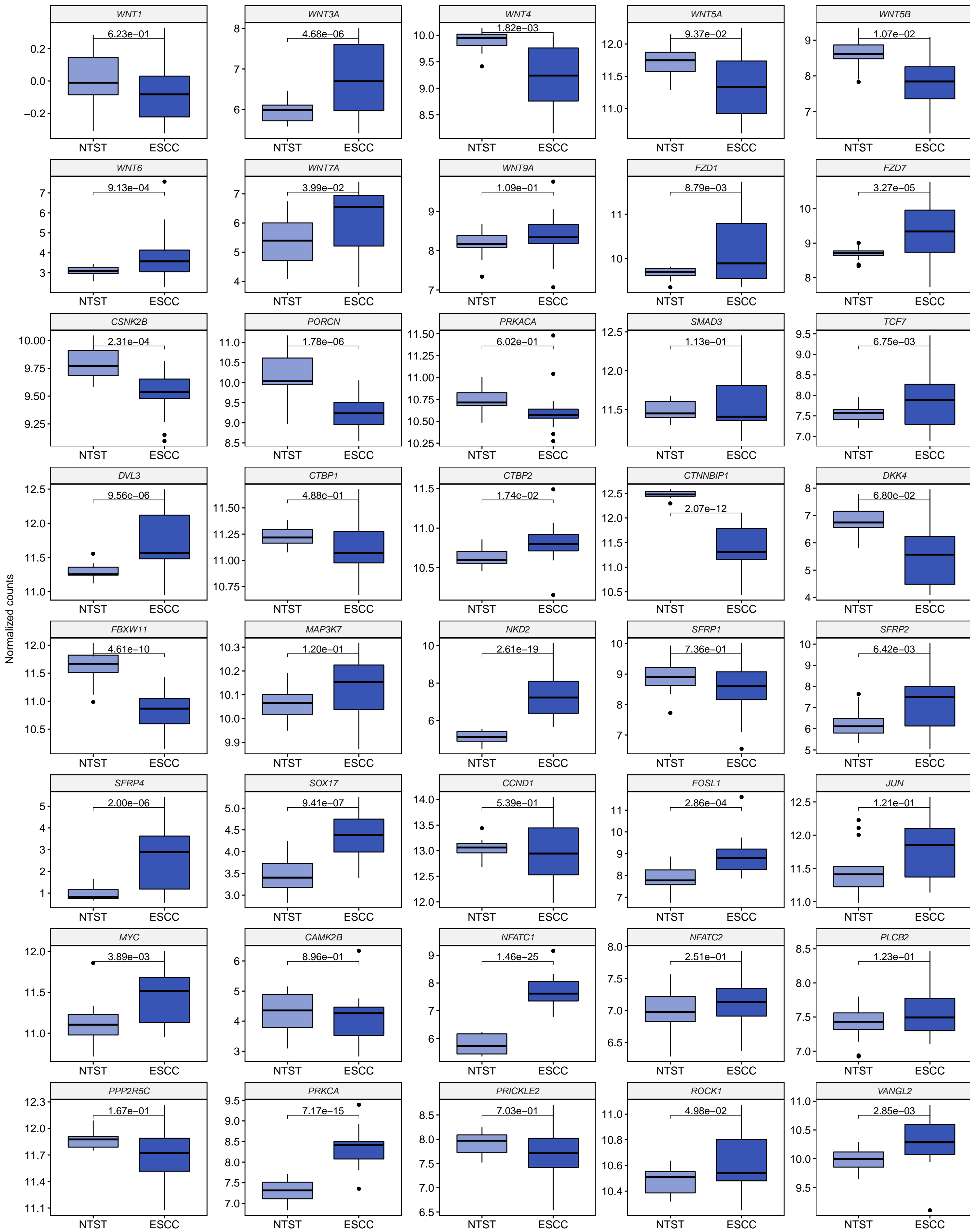

Supplement: Supplementary file 1 [file cancers-13-03014-s001.zip › Figure S5.pdf]

WNT signaling pathway

Tissue NTST LSCC

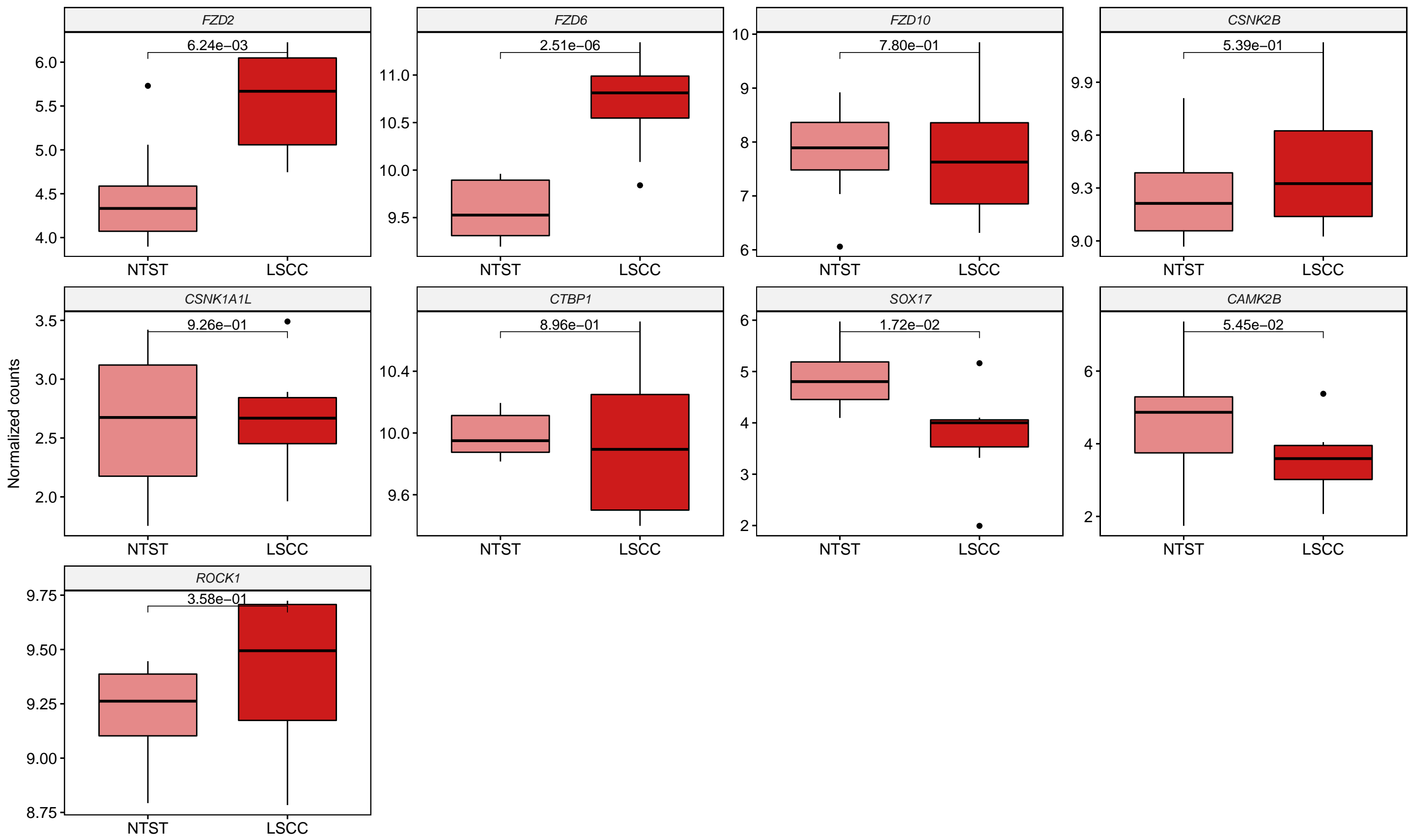

Supplement: Supplementary file 1 [file cancers-13-03014-s001.zip › Figure S6.pdf]

# ESCC-INCA

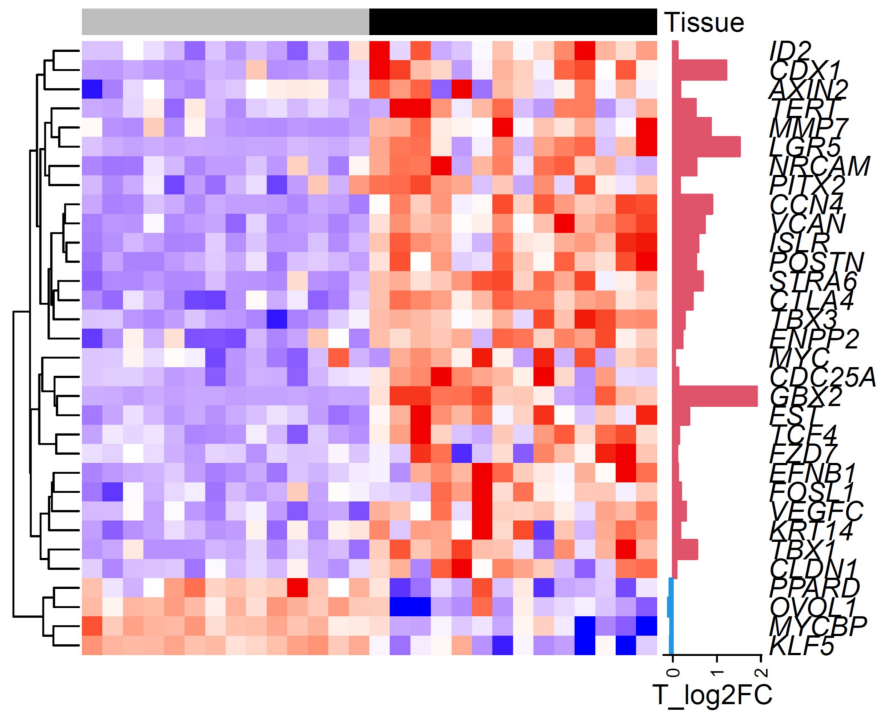

# LSCC-TCGA

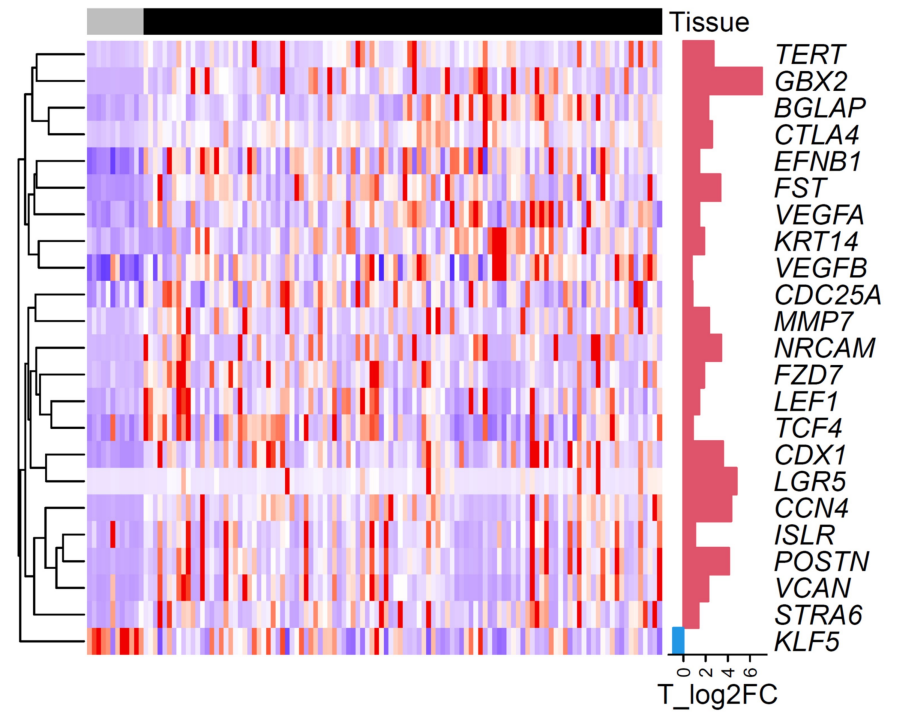

# OSCC-TCGA

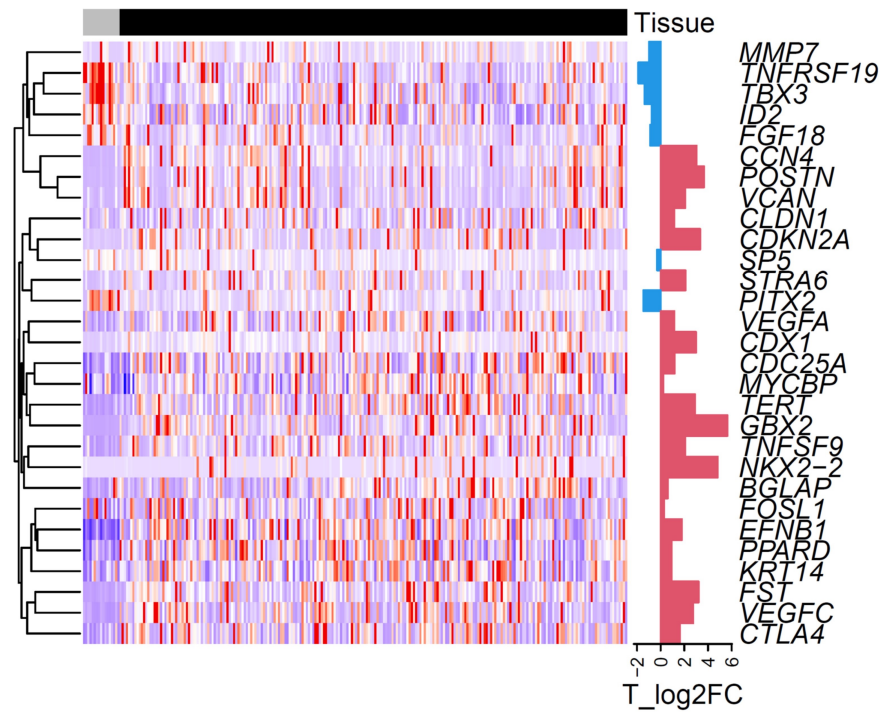

**z-score**

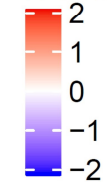

**Tissue**

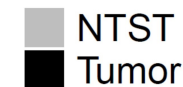

Supplement: Supplementary file 1 [file cancers-13-03014-s001.zip › Figure S7.pdf]
